# Evaluation of Alkaloids Isolated from *Ruta graveolens* as Photosynthesis Inhibitors

**DOI:** 10.3390/molecules23102693

**Published:** 2018-10-19

**Authors:** Olívia Moreira Sampaio, Lucas Campos Curcino Vieira, Barbara Sayuri Bellete, Beatriz King-Diaz, Blas Lotina-Hennsen, Maria Fátima das Graças Fernandes da Silva, Thiago André Moura Veiga

**Affiliations:** 1 Department of Chemistry, Federal University of Mato Grosso, Cuiabá-MT 78068-600, Brazil; olysampa@ufmt.br; 2 Engineering Institute, Federal University of Mato Grosso, Várzea Grande-MT 78060-900, Brazil; lucasccurcino@gmail.com; 3 Department of Chemistry, Federal University of Lavras, Minas Gerais-MG 37200-000, Brazil; barbarabellete@gmail.com; 4 Department of Biochemistry, University Nacional Autonoma de Mexico, Mexico City 04510, Mexico; kingbeat@unam.mx (B.K.-D.); blas@unam.mx (B.L.-H.); 5 Department of Chemistry, Federal University of São Carlos, São Carlos-SP 13565-905, Brazil; dmfs@ufscar.br; 6 Department of Chemistry, Federal University of São Paulo, Diadema-SP 09972-270, Brazil

**Keywords:** *Ruta graveolens*, photosystem II, Chl *a* fluorescence, Hill reaction inhibitors, acridone alkaloids

## Abstract

Eight alkaloids (**1**–**8**) were isolated from *Ruta graveolens*, and their herbicide activities were evaluated through in vitro, semivivo, and in vivo assays. The most relevant results were observed for Compounds **5** and **6**–**8** at 150 μM, which decreased dry biomass by 20% and 23%, respectively. These are significant results since they presented similar values with the positive control, commercial herbicide 3-(3,4-dichlorophenyl)-1,1-dimethylurea (DCMU). Based on the performed assays, Compound **5** (graveoline) is classified as an electron-transport inhibitor during the light phase of photosynthesis, as well as a plant-growth regulator. On the other hand, Compounds **6**–**8** inhibited electron and energy transfers, and are also plant-growth inhibitors. These phytotoxic behaviors based on acridone and quinolone alkaloids may serve as a valuable tool in the further development of a new class of herbicides since natural products represent an interesting alternative to replace commercial herbicides, potentially due their low toxicity.

## 1. Introduction

**Figure 1:**
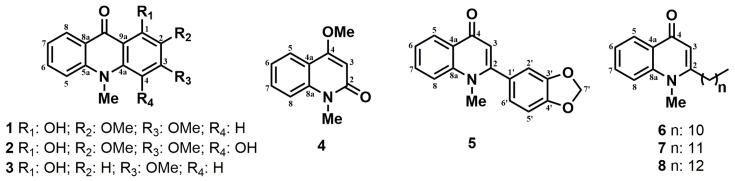
Alkaloids isolated from *Ruta graveolens*.

*Ruta graveolens* L. (Rutaceae) is a medicinal plant whose roots and aerial parts contain more than 120 special metabolites as coumarins, flavonoids, acridones, and furoquinoline alkaloids [1,2]. Many of these metabolites have attracted biological and pharmacological interest, demonstrating antifungal, phytotoxic, and antidotal activities [3,4,5,6,7,8,9]. In this context, the effect of the natural products as photosynthesis inhibitors has been efficiently evaluated [10,11,12]. The photosynthetic process is divided into three parts: the initial light-harvesting process and local charge separation, proton-coupled electron transfer, and multielectronic redox catalysis [13]. During the phenomenon, light absorption by antenna molecules is followed by efficient charge separation across the membrane via photosynthetic reaction centers [14]. The antenna system absorbs and converts light into chemical energy at P_680_. Accordingly, charge recombination is prevented by the presence of an electron-transport chain driving electrons towards P_700_; a second light-harvesting process occurs at photosystem I (PSI), providing additional energy to electrons for their final purpose: production of adenosine triphosphate (ATP) and dihydronicotinamide-adenine dinucleotide phosphate (NADPH), which are used for CO_2_ fixation through the Calvin cycle (biochemistry phase) [13,14]. Therefore, we analyzed chlorophyll *a* fluorescence kinetic transients to verify the damage on photosynthetic apparatus, demonstrating the quantitative and qualitative effects of herbicides on both photosystems [15,16]. From this perspective, the main goal of this report was to investigate the effects of alkaloids (**1**–**8**) isolated from *Ruta graveolens* L. (Figure 1) on photosynthetic activities through polarography, chlorophyll (Chl) *a* fluorescence, and in vivo plant-growth experiments. Our results suggested that these techniques are powerful and sensitive enough to localize, in detail, the mechanisms of action related to such a complex target, photosynthesis.

## 2. Results and Discussion

### 2.1. Effect of Alkaloids ***1**–**8*** on Noncyclic Electron Transport and H^+^-ATPase Activity

**Figure 2:**
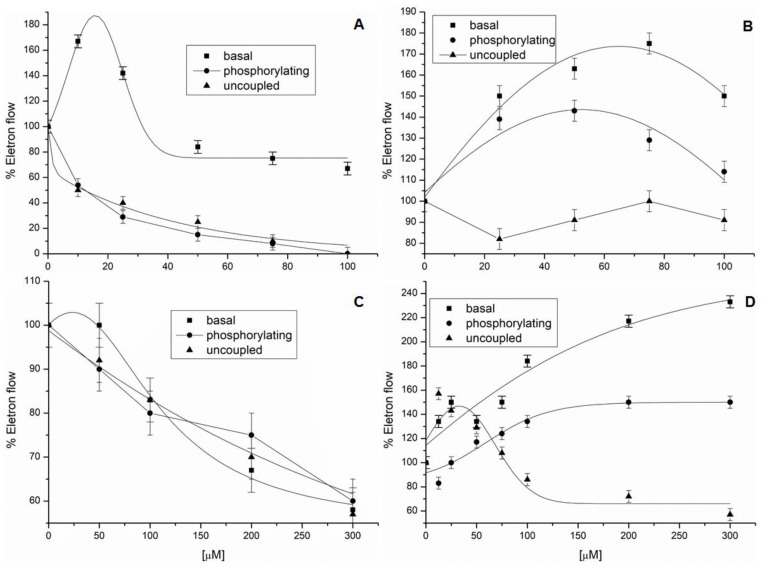
Effect of the alkaloids isolated from *R. graveolens* on electron flow. Control-rate values for electron transport from basal, phosphorylating, and uncoupled conditions were 450, 620, and 1200 µequiv e^−^ h^−1^ mg^−1^ chlorophyll (Chl)^−1^, respectively. Panel (**A**): Compound **1**; Panel (**B**): Compound **4**; Panel (**C**): Compound **5**; and Panel (**D**): Mixtures **6**–**8**.

Compounds **2** and **3** did not present an effect on noncyclic electron transport in preliminary tests. On the other hand, the other alkaloids inhibited noncyclic electron transport from H_2_O to methylviologen (MV) in chloroplasts isolated from *Spinacea oleracea* L. Arborinine (**1**) inhibited phosphorylating and uncoupled electron flow by 100% at 100 μM, which demonstrated that (**1**) behaves as a potent electron-transport inhibitor (Figure 2A). The basal electron flow was increased at low concentrations (around 15 μM), but electron flow at concentrations higher than 25 μM was decreased, inhibiting electron flow by 20% at 100 μM, which means that (**1**) binds to the CF_1_CF_0_-ATP*ase* complex, suggesting inhibitory activity on ATP synthesis. The results found, with regard to electron-transport reaction, a increment of the step as well as a decrease in the phosphorylating and uncoupled steps, indicating that (**1**) exhibited a dual effect by inhibiting both energy transfer and electron transport [17].

Compound **4** increased basal and phosphorylating electron transports by 80% and 40%, respectively, at the beginning of the illumination, and then decreased them, since the concentrations were higher than 80 μM (Figure 2B). As well as Compound (**1**), (**4**) decreased the uncoupled phase at concentrations close to 80 μM. Therefore, (**4**) did not demonstrate electron-transport inhibition, but rather acted as a decoupling agent. Graveoline (**5**) inhibited the basal, phosphorylating, and uncoupled electron transport by 40% at 300 μM, which suggested Hill reaction inhibitory behavior (Figure 2C).

Homolog mixture **6**–**8** inhibited energy transfer at 25 μM and showed slight inhibitory activity on electron-transport reactions at concentrations up to 100 μM (Figure 2D). Compounds **6**–**8** increased basal and phosphorylating electron transport by 230% and 140%, respectively. The uncoupled electron transport showed a small increase in concentrations below 100 μM. In this way, the mixture behaved mainly as an energy-transfer inhibitor and showed electron-transport inhibitory activity at higher concentrations.

When there is a significant increase on the basal electron-transport step, as observed for Compounds **1**, **4**, and **6**–**8**, this is an indication that the compounds are acting on the ATP–synthase complex [17]. Cyclic electron transport is happening normally, as can be observed in the basal reaction, due the behavior of the chloroplasts in the reaction medium. The percentage of the basal curve means that the effect is happening over the ATP–synthase complex once the basal reaction works harder to equilibrate this damage, thus increasing the speed of action. 

Due to this, ATP*ase* analysis for Compounds **1**, **4**, and **6**–**8** was needed to confirm if they interfere on the CF_1_CF_0_-ATP*ase* complex, acting by direct inhibition of ATP synthesis. The experiments (Table 1) revealed that Compound **4** binds to CF_1_CF_0_-ATP*ase* complex exerting a direct inhibition of the H^+^ gradient dissipation and the Compounds **1** and **6**–**8** act as energy transfer inhibitors (H^+^-ATP*ase* inhibitor) [17].

The electron-transport increase on the basal reaction up to 100% indicates that the compounds acted on the ATP–synthase complex, blocking the energy transfer or acting as proton-transfer decoupling. This behavior was observed for Compounds **1** and **6**–**8** through the increase of the basal step for Compound **4** by the increment of the basal and phosphorylating reactions [18,19].

To confirm if Compounds **1** and **6**–**8** act as energy-transfer inhibitors, and if Compound **4** acts as a decoupling agent, we performed H^+^-ATP*ase* assays to verify their effect on the catalytic unit of the H^+^-ATP*ase* complex (CF_0_-CF_1_) [17]. Compounds **1** and **6**–**8** inhibited the energy transfer, as they decreased the inorganic phosphate (Pi) concentrations in the reaction medium by 25% at 100 μM and 300 μM, respectively. Corroborating the electron-transport data, both compounds are inhibitors of the CF_0_-CF_1_ enzymatic site of the ATPase complex (Table 1). In its turn, Compound **4** increased Pi concentration by 18% at 100 μM, which confirmed its proton-gradient uncoupling profile. 

### 2.2. Uncoupled PSI and PSII Electron-Flow Determination

To localize the inhibition sites of the alkaloids on the thylakoid electron-transport chain, their effects on PSI and PSII (including partial reactions) were evaluated employing artificial donors and acceptors of electrons, as well as appropriate inhibitors [20]. Arborinine (**1**) inhibited uncoupled electron transport on PSII from water to DCBQ (from H_2_O to Q_B_) and the partial reactions from water to sodium silicomolybdate (SiMo) (from H_2_O to Q_A_) by 60% at 400 μM (Table 2). There were no significant results (<4%) for the reactions from DPC to 2,6-dichlorophenolindophenol (DCPIP) (from P_680_ to Q_B_).

The polarographic measures indicated that **1** inhibited the passage from H_2_O to Q_A_, that is, on both sides of the electron transport on PSII. The first inhibition site (H_2_O to SiMo) occurs in the enzyme where water photo-oxidation happens, and the other at DPC (donates electron at P680) to DCPIPox (accepts electrons at Q_B_ site), located at the water-splitting enzyme complex (OEC) and between the range of electron flow from P680 to Q_A_. These results indicated that **1** inhibited PSII at the span of electron transport from H_2_O to Q_A_ due the fact that SiMo accepts electrons exactly at the Q_A_ site. Table 2 shows that the span of electron transport from P680 to Q_B_ was not inhibited in all concentrations. Compound **1** inhibited the PSI uncoupled electron transport from reduced DCPIP to MV by 50% at 200 μM (Table 3). However, no changes were observed on inhibitory activity at higher concentrations. 

### 2.3. Chl a Fluorescence Measurements in Spinach Leaf Discs

The Chl *a* fluorescence assay is a widely used tool to evaluate the photosynthetic apparatus in plants submitted to different stresses, as well as to provide detailed information about the structure and function of PSII [10,20]. For this experiment, all alkaloids were evaluated at 150 and 300 μM. Compounds **1**–**4** showed very low activity during the experiment, less than 20% compared to negative control (data not shown).

**Figure 3:**
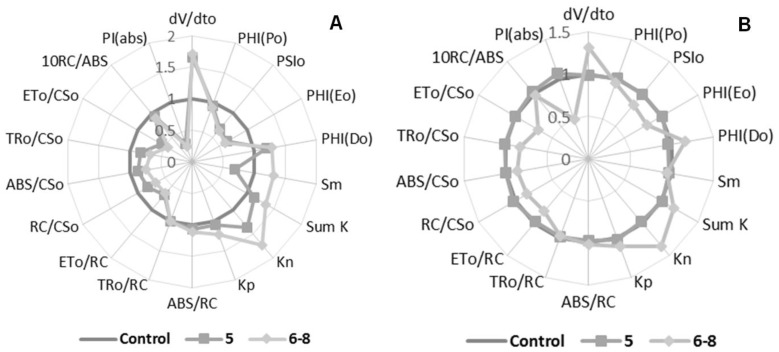
Radar plot of Compounds **5** and **6**–**8** effects on Chl *a* fluorescence parameters calculated from an *OJIP* transient curve. Panel (**A**) 150 μM, and Panel (**B**) 300 μM.

Compound **5** increased dV/dt_0_ and decreased PI*_abs_*, both parameters by 60% at 150 μM, which represents a stressful event occurring in the plant. The association of these parameters suggests that the natural redox process of photosynthesis was interfered with (Figure 3A). Parameters PSI_0_, PHI(E_0_), S*m*, ET_0_/CS_0_, and ET_0_/RC were reduced by 40%, which directly represents that electron transport on the redox process was interrupted, indicating damage to PSII. The decrease in S*m* demonstrates that not all absorbed energy was used, and then it was eliminated from the process. Energy dissipation was confirmed through the increase of the nonphotochemical “de-excitation” constant (K*n*) by 40% and the quantum yield (t = 0) of dissipation energy (PHI(D*o*)) by 20%. Thus, the energy contained in the system was released as heat or transferred to another molecule.

Compounds **6**–**8** were active at both concentrations during the leaf-disc fluorescence assay (Figure 3A,B). The PI*_abs_* parameter showed a decrease of 70% at 150 μM, indicating a nontraditional photosynthesis process. Parameters PSI_0_, PHI(E_0_), ET*_0_*/CS*_0_* and ET_0_/RC were reduced by 40, 40, 60 and 40%, respectively, at 150 μM. These decrements represent damage in electron transport on PSII, showing that the calculated quantum-yield values for the electron transport decreased in the process of the flux being inhibited. The reduction of ET_0_/CS_0_*,* ET_0_/RC, and RC/CS_0_ parameters by 30% indicated that the electron transport was being blocked, as well as a reduction in reaction centers participating in the process. Like Compound **5**, the increment was promoted by the mixture of analogs on the dV/dt_0_, Sm*K*, K*n*, and PHI(D_0_) parameters.

**Figure 4:**
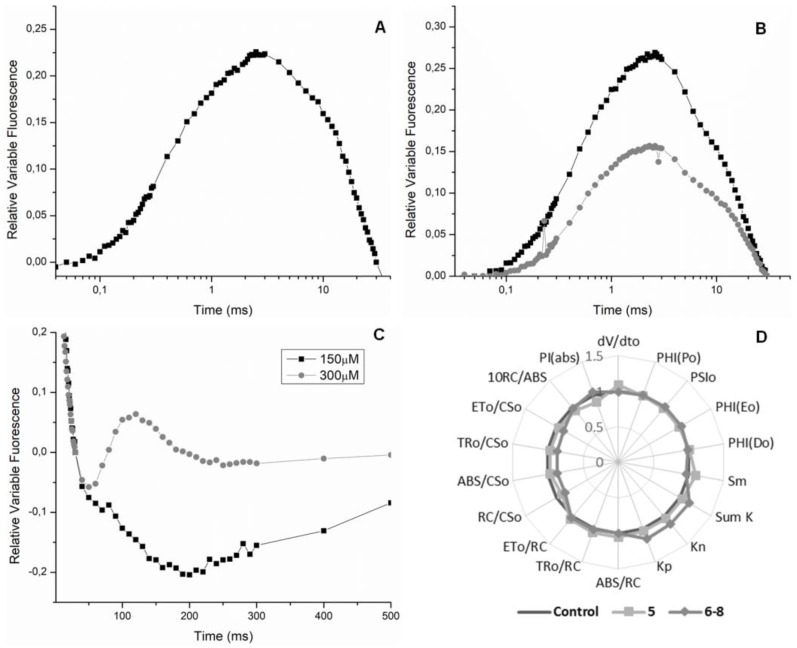
Panel (**A**) Appearance of the J-band in the presence of Compound **5** at 150 µM. Panel (**B**) Appearance of the J-band in the presence of Compounds **6**–**8** at 150 and 300 µM. Panel (**C**) Appearance of the I-band in the presence of **6**–**8** at 150 and 300 µM. Panel (**D**) Radar plot of Compounds **5** and **6**–**8** effects on Chl *a* fluorescence parameters calculated from OJIP curve of sprayed *Lolium perenne* plants after 72 h.

A *J* band (2 ms) was observed at the *OJIP* transient curve for Compound **5** (150 μM), which indicates inhibition at the quinone level, on the acceptor side of PSII (Figure 4A). An increase at *J* step can be understood as evidence for reduced-form QA accumulation (QA^−^) due electron-transport deceleration beyond QA [21]. Since the PSII electron flux was inhibited, the maximum PSII microelectrons field carries less QA^−^. This aspect corroborated the reduction of the PSI_0_ and PHI(E_0_) quantum parameters. The results of the fluorescence emission on spinach-leaf discs confirm the in vitro electron transport results, which revealed Compound **5** acting as a Hill reaction inhibitor.

The same *J* band was observed when the mixture of quinolone alkaloids was submitted to the assays (Figure 4B), which confirms that Compounds **6**–**8** also behave like 3-(3,4-dichlorophenyl)-1,1-dimethylurea (DCMU), inhibiting the acceptor side of the PSII [10]. The Chl *a* experiment also showed the appearance of the *I* band near 30 ms (Figure 4C), which exclusively refers to the efficiency of the quinone pool. This event indicates whether plastoquinones are active or not during the QA reduction process. When the *I* band is found in negative values (on the graph), it suggests that the QA pool is functioning excellently, and an increase in the *J* band is also observed, that is, this indicates that the interaction site is the reaction center (P680). The transient bands show exactly this effect (Figure 4A,B). Phase *I* appears when a dynamic equilibrium is reached between the reduction of the plastoquinone pool by the electron flow from the PSII and its oxidation due to PSI activity [22].

### 2.4. In Vivo Assays: Chl a Fluorescence Determination in Intact L. Perenne Leaves

The in vivo Chl *a* fluorescence experiment represents a powerful tool to evaluate the performance of the photosynthesis system in living plants without causing any damage to them [23]. To evaluate compound activity, solutions at 150 and 300 µM were sprayed on the leaves of *L. perenne* plants. However, only Compound **5** and the mixture **6**–**8** were tested because they presented the best results on a semivivo assay. After 24, 48, and 72 h of treatment, Chl *a* fluorescence transients were measured and the OJIP parameters were calculated employing Biolyser HP software.

Data showed that **5** and **6**–**8** on plants after 24 and 48 h were insignificant, but a small variation on photosynthetic parameters was observed after 72 h at 150 μM (Figure 4D). In short, the in vivo results are less significant than the results observed on the semivivo assay. We justify this because there are many natural obstacles that the compounds have to transcend to reach their target (the chloroplast), for example, cell walls and membranes [23].

### 2.5. Dry Biomass Determination

Dry biomass results were obtained using *L. perenne* plants 15 days after compound application. Compound **5** and the mixture **6**–**8** were evaluated at 150 and 300 μM. The other compounds were not tested, as they did not present any activity in the previously assays. DCMU, a herbicide, was used as positive control (Table 4). Fortunately, the best results were observed for the lowest concentration, 150 μM. Treatments **5** and **6**–**8** decreased dry biomass by 20% and 23%, respectively, compared to negative control. These are significant results since they behaved like DCMU, which reduced 23% of the biomass of the target plant.

Based on in vitro, semivivo, and in vivo approaches, Compound **5** acts as a photosynthetic electron-transport inhibitor and as a plant-growth regulator. Mixture **6**–**8**, on the other hand, acts as an electron-transport and energy-transfer inhibitor, as well as plant-growth regulator. Our results showed that almost all alkaloids behaved as photosynthesis inhibitors once some of them acted as Hill reaction inhibitors. Through fluorescence measurement, we could observe the presence of transient bands *J* and *I* (obtained from *OJIP*-test). These steps suggest that compounds isolated from *R. graveolens* inhibited electron flow on the acceptor side of PSII, exactly like DCMU does. Therefore, the aim of our work was to present that natural products still could be employed on programs to lead to new scaffold models for herbicides in the future, since natural products remains an interesting alternative to replace the commercial herbicides.

## 3. Materials and Methods

### 3.1. Alkaloid Isolation from Ruta Graveolens

The ethanolic extract (203.6 g) from *Ruta graveolens* leaves was solubilized in CH_3_OH:H_2_O (1:3, *v*:*v*) and extracted by liquid–liquid partition with hexane and dichloromethane to obtain the partitioned extract fractions. 

The dichloromethane fraction (14.6 g) from *Ruta graveolens* leaves was subjected to a chromatographic column using silica gel 60 (70–230 mesh), employing as a mobile phase increasing hexane, dichloromethane, acetone, and methanol concentrations to obtain 6 fractions (1–6). Fraction 5 (0.746 g) was subjected to a new chromatographic procedure using Sephadex LH-20 with isocratic elution formed by dichloromethane:methanol (1:1, *v*:*v*) to afford arborinine (**1**, 18.4 mg) and 1,4-dihydroxy-2,3-dimethoxy-*N*-methylacridone (**2**, 16.5 mg) [24]. 

1-hydroxy-3-methoxy-*N*-methylacridone (**3**, 17.9 mg) was obtained from the dichloromethane:hexane fraction of *Ruta graveolens* leaves using silica gel (70–230 mesh) and solvents of increasing polarity (hexane, dichloromethane, acetone, and methanol), followed by a second chromatographic purification over Sephadex LH-20 with isocratic elution dichloromethane: methanol (1:1, *v*:*v*) [25].

From the methanol fraction of *Ruta graveolens* leaves, the *N*-methyl-4-methoxy-2-quinolone (**4**, 19.3 mg) and graveoline (**5**, 14.1 mg) compounds were purified using a chromatographic column with silica gel as support, and hexane, dichloromethane, acetone, and methanol as the mobile phase [26].

The ethanolic extract (7.0 g) from the *Ruta graveolens* roots was solubilized with methanol:water (1:3 *v*:*v*), and subjected to liquid–liquid extraction with hexane to provide the respective fraction (1.53 g). The hexanic fraction was subjected to purification using silica gel (70–230 mesh). The mobile phase was composed of increasing portions of hexane, dichloromethane, acetone, and methanol to obtain 8 fractions (I–VIII). Fraction II (0.105 g) was subjected to new chromatographic purification by Sephadex LH-20 with isocratic elution dichloromethane:methanol (3:7, *v*:*v*) to obtain a homolog mixture of **6**, **7**, and **8**. The mixture was analyzed with GC-MS. The instrument was set to an initial temperature of 150 °C, and maintained at that temperature for 1 min. At the end of this period, the oven temperature was increased to 300 °C, at the rate of 10 °C/min, and maintained for 20 min. The chromatogram presented 3 peaks at retention times (t_R_) 11.5 min (**6**, *m*/*z* 313), 12.0 min (**7**, *m*/*z* 327), and 13.0 min (**8**, *m*/*z* 341). Based on the GC-MS experiment, a ratio of 8:1:1 (based on the peak areas) was estimated for **6**, **7**, and **8 [27]**.

**Compound 1.**^1^H-NMR (200 MHz, CDCl_3_) δ: 3.81 (s, 3H, *N*-Me), 3.92 (s, 3H, 3-OMe), 4.00 (s, 3H, 2-OMe), 6.23 (s, 1H, H-4), 7.23 (ddd, *J* = 8.0, 6.8 and 0.7 Hz, 1H, H-7), 7,50 (dl, *J* = 8.0 Hz, 1H, H-5), 7.73 (ddd, *J* = 8.0, 6.8 and 1.4 Hz, 1H, H-6), 8.42 (dd, *J* = 8.0 and 1.4 Hz, 1H, H-8), 14.75 (s, 1H, OH). ^13^C-NMR (100 MHz, CDCl_3_) δ: 34.1 (*N*-Me), 56.0 (C3-OMe), 60.8 (C2-OMe), 86.8 (C-4), 105.8 (C-9a), 114.5 (C-5), 120.8 (C-8a), 121.5 (C-7), 126.2 (C-8), 130.2 (C-2), 134.6 (C-6), 140.5 (C-4a), 142.0 (C-5a), 156.2 (C-1), 159.3 (C-3), 180.8 (C-9).

**Compound 2.**^1^H-NMR (200 MHz, CDCl_3_) δ: 3.96 (s, 3H, *N*-Me), 3.99 (s, 3H, 2-OMe), 4.03 (s, 3H, 3-OMe), 7.32 (ddd, *J* = 8.0, 7.5 and 1.6 Hz, 1H, H-7), 7.48 (dl, *J* = 8.7 Hz, 1H, H-5), 7.76 (ddd, *J* = 8.7, 7.5 and 1.6 Hz, 1H, H-6), 8.36 (dd, *J* = 8.7 and 1,6 Hz, 1H, H-8), 14.69 (*s*, 1H, 1-OH). ^13^C-NMR (50 MHz, CDCl_3_) δ: 44.0 (*N*-Me), 61.0 (C2-OMe), 61.5 (C3-OMe), 109.4 (C-9a), 116.6 (C-5), 121.3 (C-8a), 122.1 (C-8), 126.2 (C-7), 134.6 (C-6), 134.7 (C-2), 140.0 (C-3), 146.1 (C-5a), 151.5 (C-4), 155.8 (C-1), 157.0 (C-4a), 182.3 (C-9).

**Compound 3.**^1^H-NMR (200 MHz, CDCl_3_) δ: 3.77 (s, 3H, *N*-Me), 3.90 (s, 3H, OMe), 6.30 (s, 2H, H-2 and H-4), 7.30 (ddd, *J* = 8.0, 7.2 and 1.6 Hz, 1H, H-7), 7.40 (dl, *J* = 8.0 Hz, 1H, H-5), 7.73 (ddd, *J* = 8.0, 7.2 and 1.6 Hz, 1H, H-6), 8.44 (dd, *J* = 8.0 and 1.6 Hz, 1H, H-8), 14.75 (s, 1H, OH), ^13^C-NMR (50 MHz, CDCl_3_) δ: 33.3 (*N*-Me), 55.6 (OMe), 90.1 (C-4), 94.1 (C-2), 105.0 (C-9a), 114.4 (C-5), 121.0 (C-8a), 121.4 (C-7), 126.7 (C-8), 134.1 (C-6), 142.0 (C-5a), 144.0 (C-4a), 166.0 (C-1), 166.1 (C-3), 180.0 (C-9).

**Compound 4.**^1^H-NMR (200 MHz, CDCl_3_) δ: 3.64 (s, 3H, *N*-Me), 3.92 (s, 3H, OMe), 6.23 (s, 1H, H-3), 7.96 (dd, *J* = 8.0 and 1.5 Hz, 1H, H-5), 7.21 (ddd, *J* = 8.0, 7.1 and 1.5 Hz, 1H, H-6), 7.34 (dl, *J* = 8.0 Hz, 1H, H-8), 7.58 (ddd, *J* = 8.0, 7.1 and 1.5 Hz, 1H, H-7). ^13^C-NMR (50 MHz, CDCl_3_) δ: 28.8 (*N*-Me), 55.3 (OMe), 96.1 (C-3), 113.8 (C-8), 116.2 (C-4a), 121.4 (C-6), 123.1 (C-5), 131.0 (C-7), 139.4 (C-8a), 162.4 (C-4), 163.6 (C-2).

**Compound 5.**^1^H-NMR (400 MHz, CDCl_3_) δ: 3.59 (s, 3H, *N*-Me), 5.99 (s, 2H, H-7′), 6.26 (s 1H, H-3), 6.78 (dd, *J* = 1.6 and 0.4 Hz, 1H, H-2′), 6.81(dd, *J* = 8.0 and 1.6 Hz, 1H, H-6′), 6.84 (dd, *J* = 8.0 and 0.4 Hz, 1H, H5′), 7.34 (ddd, *J* = 8.0, 6.8 and 1.6 Hz, 1H, H-6), 7.49 (dl, *J* = 8.4 Hz, 1H, H-8), 7.64 (ddd, *J* = 8.4, 6.8 and 1.6 Hz, 1H, H-7), 8.37(dd, *J* = 8.0 and 1.6 Hz, 1H, H-5).

**Compounds 6**–**8**. ^1^H-NMR (400 MHz, CDCl_3_) δ: 0.87 (t, *J* = 8.0 Hz, 3H, CH_3_-9′), 1.26–2.37 (2H-2′ to 2H-8′, overlapping with the signals of **7** and **8**), 3.05 (qt, *J* = 8.0 Hz, 2H, H-1′), 4.12 (s, 3H, *N*-Me), 6.70 (s, 1H, H-3), 7.54 (tl, *J* = 8.0 Hz, 1H, H-8), 7.77 (tl, *J* = 8 Hz, 1H, H-6), 8.19 (tl, *J* = 8.0 Hz, 1H, H-5), 8.19 (tl, *J* = 8.0 Hz, 1H, H-7).

### 3.2. Chloroplast Isolation and Chlorophyll Quantitative Determination

Intact chloroplasts were isolated from spinach leaves (*Spinacea oleracea* L.), as previously described [12,22,25]. Chlorophyll concentration was measured spectrophotometrically through a chloroplast suspension in a solution of 400 mM sucrose, 5 mM MgCl, 10 mM KCl, 30 mM tricine-KOH, and pH 8.0 [12].

### 3.3. Measurement of Noncyclic Electron Transport Rate

The light-induced noncyclic electron-transport activity from water to MV was determined polarographically employing a Clark-type electrode in the presence of 50 μM of MV [19]. Basal electron transport was quantified by illuminating a solution of chloroplasts (20 μg Chl/mL) in 3 mL of 100 mM sorbitol, 10 mM KCl, 5 mM MgCl_2_, 0.5 mM KCN, 15 mM tricine-KOH, and 50 μM MV at pH 8.0 for 1 min. The phosphorylating electron-transport rate was estimated for the basal electron transport from water to MV, adding 1 mM of ADP and 3 mM KH_2_PO_4_. In turn, uncoupled electron transport was evaluated in the same solution used for basal step, with 6 mM NH_4_Cl added as an uncoupler [12]. 

### 3.4. Uncoupled PSI and PSII Electron-Flow Determination

Electron-flow activities were monitored by an oxygen monitor yellow spring instrument model 5300A using a Clark-type electrode. All reaction mixtures were illuminated with filtered light (5 cm filter of 1% CuSO_4_ solution) from a projector lamp (GAF 2660) at room temperature. For each reaction, a blank experiment was performed with chloroplasts in the reaction medium. Uncoupled PSII from H_2_O to DCPIP was measured through the reduction of DCPIP-supported O_2_ evolutions, monitored polarographically. The reaction medium for assaying PSII activity was composed by the same basal electron-transport medium, but in the presence of 1 μM 2,5-dibromo-3-methyl-6-isopropyl-1,4-*p*-benzoquinone (DBMIB), 100 μM DCPIP, and 300 μM K_3_[Fe(CN)_6_] and 6 mM NH_4_Cl [28].

To determine the uncoupled partial reaction of PSII from water to SiMo, solutions of 200 μM of SiMo and 10 μM of DCMU were added to the solution used for the PSII reactions (3 mL), then chloroplasts (20 μg Chl/mL) were added and illuminated for 1 min [29]. 

Uncoupled PSI electron transport from the reduced DCPIP with sodium ascorbate to MV was determined in a similar form in a basal noncyclic electron-transport medium. However, the following reagents were added: 10 μM DCMU, 100 µM DCPIP, 50 μM MV, 300 μM sodium ascorbate, and 6 mM NH_4_Cl [30]. All measurements were performed in triplicate and compared to negative control (solvent, dimethyl sulfoxide (DMSO)).

### 3.5. H^+^-ATPase Activity Measurements

Intact chloroplasts isolated from *S. oleracea L.* were resuspended in a solution of 0.35 M sorbitol, 2 mM EDTA, 1 mM MgCl_2_.6H_2_O, 1 mM MnCl_2_, and 50 mM Hepes medium at pH 7.6. H^+^-ATPase activity was measured as reported [24]. NH_4_Cl and DMSO were employed as positive and negative controls, respectively. Pi was quantified using a UV spectrophotometer with measurements in λ = 660 nm.

### 3.6. Chlorophyll A Fluorescence Measurements in Spinach-Leaf Discs

Ten 7 mm leaf discs were placed in Petri dishes with 10 mL of a modified Krebs medium containing 115 mM NaCl, 5.9 mM KCl, 1.2 mM MgCl_2_, 1.2 mM KH_2_PO_4_, 1.2 mM Na_2_SO_4_, 2.5 mM CaCl_2_, and 25 mM NaHCO_3_ (pH 7.4). The Petri dishes were maintained in orbital stirring for 12 h at room temperature. All alkaloids, **1**–**8**, were added to the system for a new period of stirring (12 h). The discs were dark-adapted for 30 min and chlorophyll *a* fluorescence was measured at room temperature through a Hansatech Fluorescence Handy PEA (Plant Efficiency Analyzer, King’s Lynn, UK) [16,25].

### 3.7. Plant Material for In Vivo Assays 

A suspension of *Lolium perenne* seeds prepared with 10% sodium hypochlorite solution was kept in an orbital shaker for 15 min. Then, the sodium hypochlorite solution was removed and the seeds were washed 3 times with distilled water; 100 seeds were placed in 12 cm diameter pots containing a mixture of 50:25:25 (*w*/*w*/*w*) soil/peat-moss/agrolite. All pots were watered daily and maintained in a greenhouse at 25–30 °C under normal day/night illumination (12/12 h). *L. perenne* plants were selected by uniformity after being 15 days old. The plants were separated in 3 groups: negative control (DMSO), positive control (50 µM of DCMU), and plants treated with each alkaloid at 150 and 300 µM [12] by being manually sprayed. 

### 3.8. Chlorophyll a Fluorescence Determination in Intact L. Perenne Leaves and Dry Biomass Determination

Chl *a* fluorescence was measured in leaves from the control plants and those treated with alkaloids **1**–**8** at 150 and 300 µM. After 24, 48, and 72 h of spraying, the leaves that adapted to the dark for 15 min were excited by light from an array of 3 light-emitting diodes delivering 3000 µmol m^−2^ s^−1^ of red light (650 nm). The Chl *a* fluorescence induction curves were measured at room temperature with a portable Hansatech Fluorescence Handy PEA apparatus. Photosynthetic parameters like as PI*_abs_*, dV/dt_0_, Sm, ABS/RC, TR_0_/RC, ET_0_/RC, TR_0_/ABS, ET_0_/TR_0_, ET_0_/ABS, PHI(D_0_), ABS/CS_0_, TR_0_/CS_0_, ET_0_/CS_0_, *k*p, *k*n, and Sum*k* were represented in a radar plot [12]. For the dry-biomass experiment, 15 days old *L. perenne* plants treated with alkaloids **1**–**8** at 150 and 300 µM were dried in an oven at 65 °C to reach a constant weight. Then, the dry biomass was measured using analytical balance [12]. 

## Figures and Tables

**Table 1:** Effect of Compounds **1**, **4**, and **6**–**8** on inorganic phospate (P*i*).

Compound	(μM)	P*i* (%)
Control	0	100
**1**	25	90
	50	78
	100	77
**4**	25	104
	50	108
	100	118
**6**–**8**	100	93
	200	88
	300	77

**Table 2:** Effects of arborinine (**1**) on photosynthetic electron transport on photosystem II (PSII). Note: DCPIP, 2,6-dichlorophenolindophenol.

(μM)	H_2_O to DCBQ		H_2_O to Sodium Silicomolybdate (SiMo)		DPC to DCPIP	
	*a*	*b*	*A*	*b*	*C*	*b*
0	547.5 ± 2.74	100	511.0 ± 2.56	100	256.0 ± 1.28	100
50	-	-	-	-	283.0 ± 1.42	110.6
100	401.5 ± 2.00	74	328.5 ± 1.64	65	268.0 ± 1.34	104.5
200	292.0 ± 1.46	54	255.5 ± 1.28	50	268.0 ± 1.34	104.5
300	255.5 ± 1.28	47	237.3 ± 1.19	47	-	-
400	219.0 ± 1.09	40	219.0 ± 1.09	43	-	-

*a* (μequiv e^−^ h^−1^ mg^−1^ Chl^−1^), *b* (%), *c* (μM DCPIP_red_ mg^−1^ Chl^−1^).

**Table 3:** Effects of arborinine (**1**) on photosynthetic uncoupled electron transport at PSI.

(μM)	DCPIP_red_ a Methylviologen (MV)	
	*a*	*b*
0	1467.4 ± 7.34	100
100	867.1 ± 4.34	59.1
200	733.7 ± 3.67	50
400	667.0 ± 3.34	46

*a* (μequiv e^−^ h^−1^ mg^−1^ Chl^−1^) *b* (%).

**Table 4:** Dry biomass assay for Compounds **5** and **6**–**8**. Note: DCMU, 3-(3,4-dichlorophenyl)-1,1-dimethylurea.

Treatment	(µM)	Dry Biomass (mg)	Percentage (%)
**Control**	0	400.0 ± 2.00	100
**DCMU**	10	307.0 ± 1.54	77
**5**	150	320.0 ± 1.60	80
	300	327.0 ± 1.64	82
**6**–**8**	150	310.0 ± 1.55	77
	300	350.0 ± 1.75	87

## Abbreviations

ATP	adenosine triphosphate
Chl	chlorophyll
DBMIB	2,5-dibromo-3-methyl-6-isopropyl-1,4-*p*-benzoquinone
DCMU	3-(3,4-dichlorophenyl)-1,1-dimethylurea
DCPIP	2,6-dichlorophenolindophenol
DMSO	dimethyl sulfoxide
MV	methylviologen
NADPH	dihydronicotinamide-adenine dinucleotide phosphate
Pi	inorganic phospate
PSI	photosystem I
PSII	photosystem II
RC	reaction center
SiMo	sodium silicomolybdate
